# Ingestible transiently anchoring electronics for microstimulation and conductive signaling

**DOI:** 10.1126/sciadv.aaz0127

**Published:** 2020-08-28

**Authors:** Alex Abramson, David Dellal, Yong Lin Kong, Jianlin Zhou, Yuan Gao, Joy Collins, Siddartha Tamang, Jacob Wainer, Rebecca McManus, Alison Hayward, Morten Revsgaard Frederiksen, Jorrit J. Water, Brian Jensen, Niclas Roxhed, Robert Langer, Giovanni Traverso

**Affiliations:** 1 Department of Chemical Engineering and David H. Koch Institute for Integrative Cancer Research, Massachusetts Institute of Technology, Cambridge, MA 02139, USA.; 2 Department of Mechanical Engineering, Massachusetts Institute of Technology, Cambridge, MA 02139, USA.; 3 Department of Mechanical Engineering, University of Utah, Salt Lake City, UT 84112, USA.; 4 Division of Comparative Medicine, Massachusetts Institute of Technology, Cambridge, MA 02139, USA.; 5 Global Research Technologies and Device R&D, Novo Nordisk A/S, Maaloev, Denmark.; 6 Department of Micro and Nanosystems, KTH Royal Institute of Technology, Stockholm, Sweden.; 7 Division of Gastroenterology, Brigham and Women’s Hospital, Harvard Medical School, Boston, MA 02115, USA.

## Abstract

Ingestible electronic devices enable noninvasive evaluation and diagnosis of pathologies in the gastrointestinal (GI) tract but generally cannot therapeutically interact with the tissue wall. Here, we report the development of an orally administered electrical stimulation device characterized in ex vivo human tissue and in in vivo swine models, which transiently anchored itself to the stomach by autonomously inserting electrically conductive, hooked probes. The probes provided stimulation to the tissue via timed electrical pulses that could be used as a treatment for gastric motility disorders. To demonstrate interaction with stomach muscle tissue, we used the electrical stimulation to induce acute muscular contractions. Pulses conductively signaled the probes’ successful anchoring and detachment events to a parenterally placed device. The ability to anchor into and electrically interact with targeted GI tissues controlled by the enteric nervous system introduces opportunities to treat a multitude of associated pathologies.

## Teaser

An ingestible device injects probes into stomach tissue for electrical stimulation and potential treatment of gastroparesis.

## INTRODUCTION

Neural circuits present in the gastrointestinal (GI) tract directly connect with vagal and spinal neurons (*1*, *2*), and the enteric nervous system is increasingly being recognized for its role in a broad set of pathologies. These go well beyond GI disorders (*3*), including links to neurodegenerative diseases such as Alzheimer’s, Hirschsprung’s, and Parkinson’s (*4*–*6*). Studies have shown that treatments using electrical impulses for nerve or muscle stimulation improve the quality of life and health outcomes for patients with a range of disorders (*7*, *8*). Electrical impulse deployment, however, is associated with a number of challenges including localization and prolonged, directed engagement with the targeted tissue. Electrical impulse treatments have traditionally required invasive procedures to accurately implant devices and direct current to precise locations. For example, surgically implanted gastric pacemakers, which stimulate the outer muscular layer of the stomach, demonstrated efficacy in humans for the treatment of gastroparesis (*9*), a pathology associated with slow gastric emptying. Prospective human trials with these devices showed a reduction in the number of weekly vomiting episodes per patient (*9*). Surgery, however, presents cost barriers and safety implications not present with orally administered therapies. Recent developments in miniaturized ingestible robotic systems and sensors, including research on locomotion and detection mechanisms, are pushing the boundaries for capsule-based therapeutics (*10*, *11*), but currently, these devices cannot yet successfully provide electrical stimulation in a controlled and extended manner similar to surgically implanted devices. An ingestible device that provides predictable and documentable electrical stimulation to the same location and layer of tissue in the stomach as the surgically implanted device could afford patients similar therapeutic benefits without the need for surgery. Moreover, oral electrical stimulation systems could be administered on an as-needed basis, allowing the patient to control exactly when they receive their therapy.

Ingestible electronic devices are used for a number of applications (*10*, *12*–*14*), including capturing video (*15*), tracking patient compliance (*16*), sensing chemical composition (*17*, *18*), reading internal temperature, measuring pH, and timing motility (*19*). However, such devices lack the locomotion and actuation components required for anchoring and interacting with the tissue wall (*14*). Capsule locomotion mechanisms in the GI tract using rigid legged structures, propellers, adhesive legged structures, magnetically actuated systems, and peristaltic stimulation are not designed to enable extended, autonomous, and directional tissue wall localization (*10*, *14*). In addition, ingestible robotic devices designed to interact with the tissue wall, such as ones used to take biopsies, do not have the ability to target specific tissue layers in the GI tract, cannot remain attached to the tissue, and do not have control mechanisms to prevent full thickness perforations during tissue interactions. Moreover, current devices often lack the ability to communicate that they have successfully attached to the tissue. Many ingestible devices use bulky antennas to wirelessly communicate with the patient (*14*), but this method drastically increases the volume of electronics in the pill. Other ingestible devices have used a compact form of electric field communication through the body to communicate the presence of the system (*16*), and this feature can be further expanded to allow for communication of additional information such as whether a device is in contact with tissue.

**Fig. 1:**
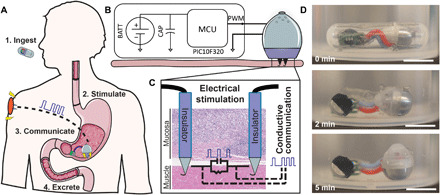
Self-orienting technology for injection and electrical microstimulation (STIMS). (**A**) STIMS injects electrically conductive hooked needles into the tissue for retention and uses them to stimulate the tissue via timed electrical pulses. The pulses also allow the device to communicate conductively to the subcutaneous space of the animal and convey proper device attachment and excretion. (**B**) STIMS architecture. BATT, Battery; CAP, capacitor; MCU, microcontroller unit; PWM, pulse width modulation. (**C**) Only the tips of the probes are conductive, which allows the electrical pulses to target a specific layer of tissue. Photo credit: Alex Abramson, MIT. (**D**) STIMS fits inside a 000 capsule, which is 26.1 mm in length and 9.9 mm in diameter, to aid in ingestion. The device includes a self-orienting system and autonomous injection mechanism, a microcontroller for pulse generation, and two coin batteries for power. A STIMS capsule was placed in 35° to 45°C water under agitation, and the device self-oriented after being released. Scale bars, 1 cm. Photo credit: Alex Abramson, MIT.

Here, we report an ingestible electronic capsule with the capacity to independently localize to the stomach wall, inject electrically conductive needle probes into the stomach lining, and remain attached to the tissue while the stomach is not digesting food, as is the case with a patient suffering from gastroparesis (Fig. 1). We show that these probes can deliver timed electrical pulses to the tissue, resulting in muscle contractions, and demonstrate that the probes can use conductive signaling to relay whether they have successfully inserted into the tissue. To ensure that the needles do not generate a complete thickness perforation during injection, we conducted an analysis of the mechanics associated with needle insertion into GI tissue. In addition, we developed optimized arrays of hooked needles that acted as anchors and provided a retentive force for the device to overcome gastric fluid flow and movement in a sedated stomach but allowed the device to release after gastric emptying began. We performed several experiments on stomach tissue both in vivo and ex vivo to determine the penetration force and needle displacement length required to achieve both a hooking and a stimulation effect from the probes. We then dosed the optimized device to swine and demonstrated the system’s ability to actuate, anchor, stimulate, and conductively communicate within a large animal model.

## RESULTS

### Device design

The self-orienting technology for injection and electrical microstimulation (STIMS) has a shape and density distribution described in our previous paper (*20*), which allows it to consistently align its injection mechanism with the tissue wall. Briefly, the device has a weighted bottom and high curvature geometric design such that its center of mass reaches a local or global minimum in exactly one orientation. In over 100 trials both ex vivo and in vivo, the self-orienting and actuation aspects of the system allowed the device to properly align with the tissue wall regardless of auxiliary attachments such as batteries and a printed circuit board (Fig. 1D and movies S1 and S2). Self-orienting occurred even when the models underwent simulated ambulation. Following endoscopic administration through an overtube into the stomachs of sedated swine, devices first localized to the lower curvature of the stomach, because of the gravitational forces felt by the device, and then self-oriented (movie S2). Of note, the lower curvature of the stomach is where surgically implanted gastric pacemaker systems are implanted (*9*). Once aligned with the tissue, the device used a compressed spring held in place by a dissolvable, timed actuation barrier to propel needles into the tissue. This hydration-based actuation mechanism was tuned to release the spring approximately 5 to 15 min after the device entered the animal, therefore allowing time for the device to pass through the esophagus (*21*), exit a capsule, and self-orient. The self-orienting system, when delivered into a sedated swine’s stomach via an overtube, was able to successfully deliver conductive 32-gauge needles into stomach tissue in every one of 25 replicate trials, demonstrating the reproducibility of the system. Further information on the actuation mechanism can be found in figs. S1 and S2. Following needle insertion, electrical current passed through the tissue in all trials in which the probes were connected to a battery and microcontroller, and the hooks at the ends of the probes provided the devices with a retentive force that kept them attached to the tissue wall.

### Hooked probes enable device retention to the stomach wall

**Fig. 2:**
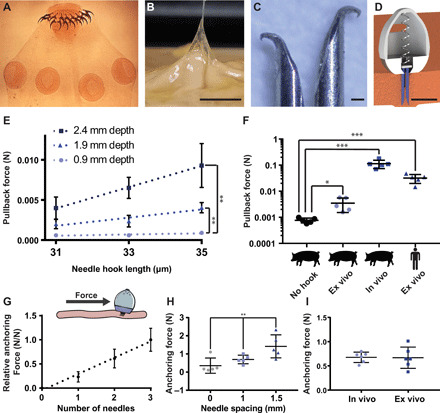
Implanted hooked needles generate retention forces that allow devices to remain localized to the stomach wall for prolonged time periods. (**A**) Scolex of the *Taenia solium* tapeworm, a parasitic worm that uses hooks to attach to the GI tract of its host. For scale, the diameter of a typical scolex for this species is 1 mm. Photo credit: Centers of Disease Control, public domain. (**B**) Hooks at the end of a 32-gauge needle as small as 30 μm in size latched onto ex vivo human stomach tissue. Scale bar, 5 mm. Photo credit: Alex Abramson, MIT. (**C**) Hooked needles fabricated in different sizes and combined into arrays created an attachment mechanism similar to the tapeworm, Scale bar, 100 μm. Photo credit: Alex Abramson, MIT. (**D**) The STIMS device inserted arrays of hooked needles into the stomach tissue. A 3D CAD model of the STIMS device, Scale bar, 5 mm. (**E**) Pullback forces from hooked needles inserted into ex vivo swine stomach (*n* = 3). (**F**) Pullback forces from hooked needles inserted into swine and human stomach tissues (*n* = 5). “No hook” data represent frictional pullback force from ex vivo swine tissue when using needles that do not hook onto the tissue. (**G**) The relative anchoring force was linearly correlated with the number of needles inserted into tissue (*n* = 9 over three stomachs). (**H**) Increasing the distance between the inserted needles increased the anchoring force (*n* = 5). (**I**) Anchoring forces were on average 0.7 N in both in vivo and ex vivo swine stomachs when using a STIMS device with three needles spaced 1 mm apart (*n* = 6 over two stomachs). Means ± SD; *P < 0.05; **P < 0.01; ***P < 0.001.

Taking inspiration from the *Taenia solium* tapeworm, a parasite that strongly latches onto GI tissue using tiny hooks (*22*), we developed a process to reliably bend the tips of the inserted probes (Fig. 2, A to F, and figs. S3 to S5) into small hooks to aid in device retention. By fixing 32-gauge stainless steel needles in a vertical position and applying a set amount of force with a steel compression platen, we created predictably sized hooks on the tips of our needles. These hooks ranged from 20 to 40 μm all the way up to 200 μm in size depending on the force applied. Hooks latched onto a layer of stomach tissue and exhibited a pullback force related to their length and depth of penetration. Needles that penetrated the tissue at least 1.9 mm demonstrated a significant pullback force (*n* = 3 per trial over three sets of trials). Swine in vivo and human ex vivo experiments with the hooks demonstrated pullback forces on the order of 0.1 and 0.05 N, respectively (*n* = 5). In addition to enhancing the needles’ ability to latch onto the tissue, the hooks also increased the force required to penetrate the tissue.

Increasing the number and spacing of needles on the STIMS device directly correlated to increased retention forces (Fig. 2, G to I). Devices resisted 0.7 N of lateral force on average when inserting three hooked needles spaced 1 mm apart into swine stomach tissue both ex vivo and in vivo (*n* = 6). Ex vivo testing in a simulated stomach environment demonstrated that devices with hooked needles remained attached to tissue for at least 7 days when exposed to a constant fluid flow of 0.1 m/s (fig. S6) (*n* = 3). This velocity is on the same order of magnitude as fluid flow in the stomach (*23*). The STIMS devices with hooked needles remained anchored to the tissue even when they were suspended perpendicular to the gravitational force, while devices without needles fell off the tissue immediately. Because the hooked needles required an actuation mechanism to initially enter the tissue, in vivo studies confirmed that the needles were not able to reenter the stomach wall after being withdrawn from the tissue.

We designed the devices to naturally detach from the tissue wall after stimulation of stomach contractions. Devices administered in vivo to swine remained in place for up to 2 hours while they were fasted and sedated even during simulated ambulation and rotation of up to 90°. However, in a separate experiment where we delivered STIMS devices to swine via an overtube and performed daily radiographs, the devices naturally detached from the stomach wall after the swine were fed a solid meal and underwent digestion between day 0 and day 1 (*n* = 9 over three swine). Of note, the devices remained in the stomach after tissue wall detachment. The swine had the ability to hold multiple devices in their stomachs at once, allowing for additional devices to be administered in the case of detachment.

**Fig. 3:**
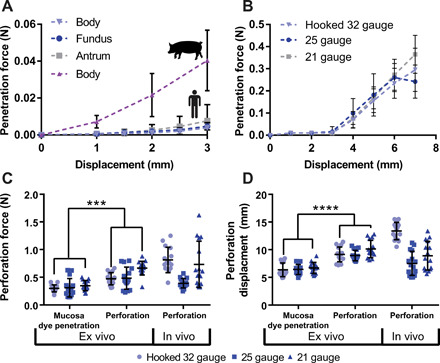
Needle insertion force profiles for human and swine stomachs. (**A**) Penetration forces in the body, fundus, and antrum of human stomachs (*n* = 9 over three stomachs) compared with penetration forces in the body of ex vivo swine stomachs (*n* = 12 over three stomachs). (**B**) Swine in vivo penetration forces with multiple sizes of needles (*n* = 15 over three stomach). (**C**) Ex vivo and in vivo perforation experiments in swine stomachs (*n* = 15 over three stomachs) showing the force and (**D**) displacement required by a needle coated in tissue marking dye to penetrate dye completely through the mucosa and to completely perforate through the muscular tissue. Means ± SD; ***P < 0.001; ****P < 0.0001.

We characterized needle penetration forces ex vivo in swine and human stomachs and in vivo in swine. The forces required to penetrate human stomach tissues from any area of the stomach were not statistically significantly different from each other (*n* = 9 over three stomachs) (Fig. 3A). In vivo swine and ex vivo human experiments demonstrated that the relationship between force and needle displacement remained similar for hooked 32-gauge, sharp 25-gauge, and sharp 21-gauge needles up until the needles began to perforate layers of the tissue (*n* = 15 over three stomachs) (Fig. 3 and fig. S7). Exact forces and needle displacements required for tissue penetration and perforation can be seen in Fig. 3 (C and D) (*n* = 15 over three stomachs). The penetration of stomach tissue proceeded through distinct penetration events: an initial event signifying penetration through superficial tissue as defined by the initial inflection point on the force-displacement characterization curve, followed by an event representing perforation of the muscular layer (fig. S8). The forces required for the initial penetration event were significantly less than those required to perforate through the entire stomach wall, thus affording the opportunity to penetrate the top layer of stomach tissue while minimizing the risk of complete perforation.

**Fig. 4:**
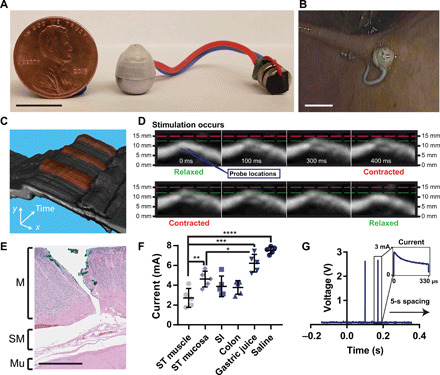
In vivo electrical stimulation of the gut. (**A**) STIMS system connected to two 1.55-V silver oxide coin cell batteries and a microcontroller. Before ingestion, electronics were coated in PDMS. Photo credit: Alex Abramson, MIT. (**B**) In vivo release and tissue localization of the STIMS. Photo credit: Joy Collins, MIT. (**C**) Stacked 2D and (**D**) sequential ultrasound images over time of muscular stimulation from electrical probes in in vivo swine stomachs. In (C), artificially colored red areas denote contraction events. MATLAB was used to isolate the muscle section from the rest of the image. A video is included in the supplement. Photo credit: Joy Collins, MIT. (**E**) Histology from ex vivo swine tissue injected by STIMS with hooked needles using a 5-N spring. Needles inserted through the mucosal layer of the stomach without perforating the tissue (M, mucosa; SM, submucosa; Mu, outer muscle). Photo credit: Kathleen Cormier, MIT. (**F**) Maximum current measured through a circuit connected in series with an in vivo swine stomach using a 2.5-V microcontroller (*n* = 5). ST, stomach; SI, small intestine. (**G**) Microcontroller stimulation events in vivo measured via an oscilloscope (*n* = 15). Scale bars, 1 cm (A and B) and 1 mm (E). Means ± SD; *P < 0.05; **P < 0.01; ***P < 0.001; ****P < 0.0001.

While the exact numbers have significant variability and fluctuate based on the dynamics of the penetration event, these results provide evidence that it is possible to target a particular tissue layer in the stomach by tuning either the force or the needle displacement. Specifically, the experimental data demonstrated that the needle required a displacement of greater than 5 mm to generate a complete thickness perforation (swine stomach tissue measures between 4 and 8 mm in thickness depending on the measurement location). Therefore, we decided to use needle displacement as a limiting measurement to ensure that the STIMS did not generate a complete thickness perforation when inserting a needle. In the STIMS device, we delivered the needles to the tissue using a compressed spring that held an excess (5 N) of force at full compression, and we limited the expansion space for the spring to 4 mm to prevent a complete thickness perforation. Histology results demonstrated that the needles from these devices passed through the stomach’s mucosa and muscularis mucosa layers into the submucosa layer of tissue adjacent to the outer muscular layer (Fig. 4E).

### Electrical stimulation induces stomach muscle contractions

Once we designed the STIMS device to safely and precisely deliver needles into the lower layers of stomach tissue, we outfitted the device with a microcontroller and a battery power source to enable electrical stimulation (Fig. 4). The Input/Output (I/O) pin of the microcontroller was connected to the stainless steel needles inside the STIMS device via stranded core electrical wires. While rigid single core wires affected the self-righting capability of the STIMS system, flexible and twistable stranded core wires allowed the device to move freely both in vitro and in vivo without significant effects from the ancillary body (Figs. 1D and 4, A and B, and movie S1). All of the electrical components were coated in a layer of polydimethylsiloxane (PDMS) to insulate them from the gastric environment. We also deposited a layer of parylene onto the shaft sections of the probes, which left only the tips of the probes conductive. This ensured that the current directly passed through the lower layer of the stomach tissue rather than being dissipated in the mucous and gastric fluid. The microcontroller generated pulse width–modulated timed electrical pulses, which enabled the device to deliver pulses to the tissue at the same frequency as surgically implanted devices currently used for gastroparesis.

In vivo testing performed in swine demonstrated that electrical pulses passed through probes appropriately placed into the stomach tissue below the mucosa had the ability to electrically stimulate and contract the muscular layer of the stomach. By attaching the parylene-coated probes to an oscilloscope, we confirmed that the pulse width modulation (PWM), controlled by the microcontroller, successfully generated electrical pulses that were conducted through the tissue. We programmed the microcontroller to deliver pulses previously demonstrated by the Food and Drug Administration (FDA)–authorized Enterra system to reduce weekly vomiting episodes in patients (*3*, *24*). Specifically, the microcontroller produced two 2.5-V square wave pulses separated 70 ms in time and each with a pulse length of 330 μs. This stimulation protocol was repeated every 5 s. The calculated charge density at the electrodes was 2.2 μC/cm^2^, an order of magnitude below the maximum charge densities recommended for stainless steel (*25*) as well as other common electrode lead materials such as titanium nitride and platinum iridium (*26*). The silver oxide batteries used had a capacity of 34 mAh at the output voltage used. Taking into account the 25-μA operating current of the microcontroller and 5-mA pulses, the device would have a total lifetime of about 1 month. In practice, the battery life may be reduced by the large current draw required for the stimulation pulses. Other gastric electrical stimulation systems with pulse lengths up to 30 ms have been shown to provide gastric pacing as well (*26*). While our battery and microcontroller can output this pulse length, it increases the electrode charge density to 200 μC/cm^2^, which is too high for our stainless steel probes to support during chronic testing. We tested our electrical setup at different locations within the stomach, small intestine, and colon, and using the 330-μs pulse lengths, our device outputted a maximum of 2 to 5 mA through the tissue depending on the tissue tested (Fig. 4F) (*n* = 5). The maximum current value was seen at the beginning of a pulse, and the current showed a decay over a single pulse, which could be due to the complex nature of heterogeneous tissue properties (Fig. 4G) (*n* = 15). There was no decay in current seen from pulse to pulse. Using an ultrasound imager, we captured muscle contractions that corresponded with an applied electrical pulse. When the probes were positioned in the muscle tissue appropriately as to provide sufficient stimulation during an electrical pulse, the tissue contracted and moved several millimeters in both the vertical and horizontal directions after receiving the pulse (Fig. 4, C and D, and movie S3). An ultrasound video of the stomach wall without electrical stimulation is shown in movie S4; in this video, no muscle contractions were observed.

### Conductive communication can signal device attachment

**Fig. 5:**
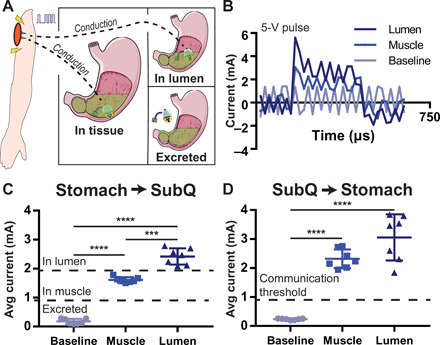
In vivo conductive communication conveys tissue wall localization. (**A**) The conductive nature of the body allows electrical pulses to pass through the tissue from the stomach to the subcutaneous space. (**B**) Current readout from a circuit branch containing a 10-ohm resistor connected to the subcutaneous space of a swine when a 330-μs, 5-V electrical pulse is generated by a microcontroller in the stomach. Electrical pulses have different profiles depending on if they originate in the stomach lumen or stomach muscle. (**C**) Average current of the pulse passing through the body in the subcutaneous space when a pulse is generated in the stomach. Depending on the current reading, it is possible to discern whether probes are in the stomach muscle, are floating in GI luminal fluid, or are not in contact with the body. (**D**) Pulses generated in the subcutaneous space can be read in the stomach as well. Means ± SD; ***P < 0.001; ****P < 0.0001.

To verify that the STIMS successfully implanted its probes into the tissue wall, we demonstrated the ability of the device to conductively communicate with separate transdermal electrodes placed on the swine’s abdomen (Fig. 5). Electrical pulses delivered in the stomach were conducted through the tissue and recorded in a parallel circuit branch just below the skin in the subcutaneous space (*n* = 7). By measuring the shunting current passing through this circuit branch, we could determine whether the probes were inserted into the tissue wall, floating in the gastric luminal fluid, or not in contact with the body at all (Fig. 5, B and C). The current passing through the subcutaneously placed probes was statistically significantly different when the STIMS probes were placed in each of these stomach locations. To accentuate the differences in current readings, we produced 5-V square waves in this experiment as compared to 2.5-V square waves used during the muscle stimulation experiments. This communication method could not be sensed by probes placed outside of the swine’s body, ensuring that the signals could not be intercepted by other devices. We also demonstrated that electrical pulses delivered into the subcutaneous space could be picked up by the STIMS probes in the stomach, allowing the system on the outside of the body to send signals to the ingested device as well (Fig. 5D). Every reading in the figures represents a different probe location in both the stomach and the subcutaneous space to ensure that the communication method was not location dependent. While the differences in current readout demonstrate some variability, averaging multiple signal readings together increases the prediction accuracy of the system. Most of the above communication, retention, and electrical stimulation experiments were performed on a swine that had undergone a laparotomy to enable oscilloscope, force gauge, and ultrasound probe access to the stomach lining, but further studies confirmed the devices’ effectiveness when administered orally.

After performing the above proof-of-concept studies, we orally delivered three STIMS systems via an overtube to swine. We confirmed that all three devices anchored into and electrically stimulated the tissue (fig. S9). Endoscopic visualization verified that the STIMS actuated correctly within 5 to 15 min after administration, delivered its three 32-gauge needles into the stomach tissue, and remained in place after simulated ambulation. The subcutaneous sensor recorded the electrical pulses produced by the STIMS devices; these pulses read out on the oscilloscope with a higher magnitude than the background noise, demonstrating that the STIMS delivered electrical current that traveled through the stomach tissue and reached the subcutaneous readout system. Last, we performed a laparotomy and measured the anchoring force of each device to be 0.78, 0.66, and 0.55 N, respectively.

## DISCUSSION

Here, we designed and tested an orally administered device that reliably injects retentive needles into the gastric tissue and provides electrical stimulation at a current, pulse width, and physical location comparable to FDA-approved surgically implanted gastric pacemakers. In vivo *s*wine studies demonstrated that devices inserted an array of hooked 32-gauge needles into the gastric submucosa, resisted gastric forces in the fasted state, provided electrical stimulation pulses to the tissue, and allowed the devices to remain attached to the tissue for several hours.

The STIMS system generally landed in the lower curvature of the stomach due to gravitational forces, the same location at which the gastric pacemaker leads are implanted. However, if a patient receiving the device were to move around during actuation, the device may attach to another area of the stomach or not attach to the tissue at all, potentially reducing the effect of the device. In this case, another device could be administered. The STIMS device could also be placed endoscopically, providing a greater control over the placement and still providing a less-invasive administration route to surgery.

We demonstrate the ability to deliver electrical pulses to the GI tissue with a maximum current of 2 to 5 mA using the STIMS. This compares to 5 mA of current used in the clinically available surgically implanted devices. When targeting tissues or tissue areas that demonstrated lower current readings, one could further increase the current delivered by optimizing the probe material and interfacial area. Increasing the current or pulse length and confirming proper tissue contact will be required to ensure that the STIMS device consistently stimulates the muscle with a strong enough signal regardless of the tissue or area of tissue targeted. While the thinness of the small intestine and colon membranes limited us from safely delivering electrical stimulation to the lower GI tract using needles, the STIMS could potentially still deliver electrical pulses using noninvasive conductive strips.

Literature on implantable gastric pacemakers suggests that the electrical stimulation therapy may work through multiple mechanisms of action in addition to directly stimulating muscle contraction including interacting with interstitial cells of Cajal, increasing gastric accommodation, changing the frequency of gastric slow waves, altering sympathovagal activities, and/or influencing central nervous system control mechanisms for nausea and vomiting (*27*). Further studies on the STIMS will be required to test for other physiological effects besides muscle stimulation.

While our stimulation device used stainless steel needles as a proof of concept, long-term electrical stimulation devices such as the FDA-approved gastric (*27*) and cardiac pacemakers (*28*) use other metals for their leads such as platinum and iridium to prevent lead corrosion and also may coat their leads in a steroid-eluting polymer to prevent tissue fibrosis. Future work should examine new lead materials that may provide greater biocompatibility. In addition, the STIMS device is a voltage-controlled system, which results in a stimulation current that depends strongly on electrical impedance. This could cause a change in applied current over long time periods over which impedance may increase due to potential fibrosis and tissue scarring. Future work should also focus on developing a current-controlled system.

Our device uses 32-gauge needles to hook onto the tissue and provide an anchoring, retentive force. Currently, STIMS uses a retention force of 0.7 N that is overcome after food consumption. Studies have shown that only 1.5 N or more of retention force allows devices to remain in the stomach after consuming a meal (*29*). The gastric-emptying force felt in the fasted state is much lower and measures around 5 mN in humans (*30*). Swine dosed during this experiment did not suffer from gastroparesis and therefore had strong gastric-emptying forces that dislodged the devices after food intake and digestion; however, our in vivo and ex vivo studies demonstrated the ability of the device to remain attached to the tissue wall in a swine stomach that was not digesting food. Studies in humans have demonstrated that even temporary electrical stimulation in the gut provides both immediate and long-lasting positive outcomes such as reduced vomiting and faster gastric-emptying times (*31*). Still, further experiments are needed to assess STIMS retention and efficacy in humans suffering from gastroparesis. While our application method did not require an on-board dislodging mechanism, methods of detachment such as dissolving needles or needle retraction mechanisms could be incorporated into the device. In particular, the microcontroller already packaged in the device could be programmed to control the device’s spring actuation and therefore the anchoring and dislodging events. Moreover, the addition of a gas sensor to the STIMS electronic architecture could be used to ensure that the device has localized itself to the stomach and not a different area of the GI tract (*17*, *32*).

By localizing to a single spot on the tissue in the absence of digestion, the device can perform a required action for an extended period of time. In fact, as research in wireless power and energy-harvesting technologies continue to progress, residence times for ingestible electronics will be limited by retention rather than power constraints (*33*, *34*). Changing the coating on the electronics from PDMS to a nonporous biocompatible plastic such as the one used in the PillCam and adding components to facilitate a high battery discharge may allow the circuit board and batteries to remain active in the gastric cavity for longer periods of time in cases where gastric emptying is not stimulated quickly.

Much of this technology is based around the careful safety analysis of delivering needles into the GI tract. Our analysis of ex vivo human tissue as well as ex vivo and in vivo swine tissue defined limits for needle force and displacement to mitigate the risk of perforation. Gastroenterologists routinely deliver needle-based injections to the stomach and intestinal walls during endoscopy using a 5-mm-long 25-gauge Carr Locke needle. During a study encompassing 1210 upper endoscopy procedures, physicians did not experience a single complication due to perforation, and perforations due to lower endoscopy occur at a rate of less than 1% (*35*, *36*). In addition, retrospective studies examining the complication rates associated with swallowed sharp objects concluded that items less than 2 cm in length provide less risk of perforation than objects greater than 2 cm in length (*37*). We have also previously shown that self-orienting devices with protruding needles safely pass through and exit the GI tract (*20*). Still, understanding the effects of force and displacement on hooked and unhooked needles of different sizes allowed us to engineer a device with the safest possible specifications.

The STIMS capacity for tissue wall anchoring and signaling could enable new applications for ingestible devices that focus on the tissue rather than the ambient environment. As the device is further developed, it may be possible to implant temporary electronic sensors into the tissue wall. Sensors that sample interstitial fluid, such as glucose sensors, currently require implantation or maintain large components outside the body, and these could be transformed into ingestible devices by using the technology presented here. In addition, as research progresses on the microbiome, it has become clear that electrical neurological signals associated with bacteria residing on the surface of the GI tract play a role in both digestion and overall health (*5*). Furthermore, mucus coatings on the tissue walls are essential in GI health. An ingestible electronic device that could reliably sample and sense variations in the mucus coating of the tissue wall could potentially allow for the diagnosis of inflammatory bowel disease, malignancy, GI infections, and several other diseases (*38*). New opportunities for electronic-based drug delivery also present themselves. For example, a STIMS device could be used as a mechanism for GI iontophoresis. Proof-of-concept studies have demonstrated that iontophoresis allows for the delivery of macromolecules via the gut, yet there are currently no ingestible devices which exist that can enable such an approach (*39*).

The STIMS device has the ability to consistently localize to the lower curvature of the stomach wall in a prescribed orientation, inject retentive and conductive probes at a set time after ingestion, and electrically stimulate the stomach tissue for prolonged periods of time for potential therapeutic benefits for patients suffering from gastroparesis. As ingestible electronic devices continue to evolve, the demand for precise and consistent localization will increase. The size and complexity of the attached electronics system, however, are limited by its size. The STIMS fits inside a 000 capsule, the largest capsule used for oral drug delivery, which measures 26.1 mm in length and 9.9 mm in diameter. Some FDA-approved ingestible electronic devices are larger than this capsule, such as capsule endoscopy systems measuring 26 mm in length and 11 mm in diameter, but capsules this size may not be suitable for regular therapy due to safety concerns associated with GI obstruction (*40*). In addition, the differences in physiology between swine and humans require additional research to ensure the safe delivery of needles into the GI tract. In particular, while we demonstrated the ability of the device to fit inside an ingestible capsule, we anesthetized our animal model and used an overtube to pass the device from the oral cavity through the esophagus and into the stomach because swine tend to chew on items before swallowing. Moreover, given the size of the oscilloscope and ultrasound equipment used to measure critical device functionalities, some of the experiments were required to be performed under a laparotomy, a technique that maintains a close, but not exact, approximation to delivery via an overtube. Future work involving the implementation of new functionalities will help to further realize the potential for combining self-orientation, implantation, and electronics. This work should focus on packaging the electronics in new ways to take advantage of the orientation specificity inherent within the system and unlock new therapeutic and diagnostic tools for clinicians.

## MATERIALS AND METHODS

Three-dimensional (3D) printed materials were designed in Solidworks (Dassault Systemes, Velizy-Villacoublay, France) and printed on a Form 2 stereolithography 3D printer (Formlabs, Somerville, USA). Polycaprolactone (PCL) with a molecular weight of 45,000 was purchased from Sigma-Aldrich (St. Louis, USA). Isomalt was purchased from Biosynth (Itasca, USA). Swine tissue for ex vivo evaluation was procured from the Blood Farm Slaughterhouse (West Groton, USA). Human stomach tissue was acquired through the National Disease Research Interchange (Philadelphia, USA). BD precision glide needles were procured from Becton Dickenson (Franklin Lakes, USA), except for nonsurface-modified 32-gauge needles, which were supplied by Novo Nordisk (Hillerod, Denmark). A Nyquist plot for 304 stainless steel, the material used for the needle probes, can be found here (*41*). Tissue marking dye was obtained from Cancer Diagnostics Inc. (Durham, USA). Female Yorkshire swine used for in vivo experiments were acquired from Tufts University (Grafton, USA). Standard 000 hypromellose Vcap capsules were acquired from Capsugel (Morristown, USA).

All animal experiments were preapproved and conducted in accordance with protocols set forth by the Massachusetts Institute of Technology (MIT) Committee on Animal Care. All of the studies performed on human tissue were reviewed by the MIT Institutional Review Board, the Committee on the Use of Humans as Experimental Subjects, and received an exempt status.

### Device fabrication and in vivo testing

The self-orienting and actuation portion of the STIMS design can be seen in fig. S1. A two-part negative mold was 3D printed for the STIMS’s top portion. The top portion was fabricated by melting PCL and casting it into the two-piece negative mold. PCL tops were used during ex vivo retention trials. In vivo tops were either fabricated from SABIC polypropylene 58MKN-10 (Riyadh, Saudi Arabia) via injection molding on a DEMAG Ergotech Pro 25-80 (Struer, Denmark) or were fabricated using a Projet 6000HD SLA printer (3D systems, Rock Hill, USA) and VisiJet SL Flex ink (3D systems) at Novo Nordisk’s Device Department (Hillerod, Denmark) with an externally evaluable actuation marker for monitoring of successful deployment. The bottom portion of the device, which was composed of 304 stainless steels, was fabricated on a DMG eco-turn 310 turning machine (Bielefeld, Germany) at Novo Nordisk’s Device Department. Each self-orienting device weighed 0.77 g, with 12% of the weight attributed to PCL and the rest of the weight attributed to stainless steel. Self-orienting devices used in vivo had a 15 to 25% larger radius and were 35 to 50% heavier than those used ex vivo.

Once the top and bottom portions of the self-orienting devices were fabricated, a custom-made spring (*k*, 0.55 N/mm; free length, 10.9 mm; compressed length, 1.75 mm; diameter, 2.2 mm) procured from Novo Nordisk was attached to the top section using PCL. The needles were then attached to the free end of the spring using a custom 3D-printed needle holder.

Prior to incorporating the needles onto the device, needles were connected to flexible wires using a conductive epoxy or solder (McMaster). The wires pictured in the figures were 26-gauge StriveDay silicone-coated stranded core wires, but 36-gauge silicone-coated stranded core wires were also used. The shafts of the 32-gauge needles were then coated in parylene C (Specialty Coating Systems, Indianapolis, USA) using a PDS 2010 Labcoter 2 (Specialty Coating Systems), while the tip sections were left bare. This allowed the probes to preferentially conduct an electrical signal though the lowermost tissue layers. The thinner wires provided the self-orienting device with a greater freedom of movement and also allowed the entire device to fit more easily inside a 000 capsule. After attachment to the device, the wires were fed out from a small hole in the system and attached to the microcontroller and battery circuit described in the “In vivo testing to verify electrical stimulation” section.

Isomalt was identified as an optimal material with brittle fracture mechanics to serve as the humidity-sensitive sensor and actuator given its known dissolution parameters within minutes (*42*) to provide actuation aligned with esophageal transit. Isomalt was molded into either a 1-mm-thick pellet or a 7-mm-diameter dissolvable disk (fig. S2) either via compression or by heating to 210°C. The actuation mechanism was positioned in the device to hold the compressed spring in place. For STIMS devices delivered via an overtube, the pellet was placed in the hole at the top of the device and rested in the needle holder, enabling visualization by the endoscope to confirm device actuation. Last, the top and bottom portions were compressed together and sealed via a rotational locking mechanism.

Experiments testing the ability of the assembled systems to function in vivo were performed in sedated swine. We placed the swine on a liquid diet for 24 hours and fasted the swine overnight prior to performing our experiments. We sedated female swine (65 to 85 kg) with intramuscular injection of Telazol (tiletamine/zolazepam) (5 mg/kg), xylazine (2 mg/kg), and atropine (0.05 mg/kg). Animals were intubated and maintained under anesthesia with isoflurane (1 to 3% in oxygen). After completing the experiments, the swine were euthanized with sodium pentobarbital at 80 mg/kg IV. Some devices were endoscopically administered via an overtube, and visualization via the endoscopic camera system allowed us to confirm self-orientation and needle actuation. Radiographs on the animals were performed immediately following administration, 2 hours after administration while remaining under sedation, and daily until the devices detached from the stomach tissue. Device detachment was confirmed by comparing the current device location to its original location in the swine stomach on the radiograph image. It was not possible to perform continuous subcutaneous monitoring of the device attachment, as the equipment required for this test could not be safely introduced into the swine habitat. Moreover, it was not possible to sedate the swine several times per day to perform radiographs, as this would be harmful for the animals. It was not possible to orally administer the devices to awake animals, as swine regularly chew what they ingest. Moreover, endoscopic administration allowed us to maintain a constant visualization of the system as compared with solely administering the device via an overtube. However, we demonstrate in Fig. 1 the ability of the device to be encapsulated in a 000 capsule (a capsule approved for oral administration in humans), we demonstrate the ability of the device to exit the capsule and self-orient ex vivo, and we demonstrate the ability of the device to function fully after administration freely via an overtube. Of note, it was not possible to confirm the exact device retention force or electrical stimulation provided to the nearby stomach tissue by the system without performing a laparotomy, as such experiments required large pieces of equipment that could not access the stomach via an overtube. Detailed protocols used to confirm these aspects of the system can be found in the following Materials and Methods sections: “Tissue retention experiments”, “In vivo testing to verify electrical stimulation”, and “Conductive communication.” Unless stated in the paper, devices were not delivered via an overtube during experiments where a laparotomy was performed. During these experiments, devices were placed on the tissue to position them in an easily accessible location for the measurement equipment.

### Hooked needle fabrication

We evaluated the forces required to create hooks of varying lengths at the tips of 32-gauge needles by fixing such needles to an Instron 5943 machine (Instron, Norwood, USA) and compressing them with a hardened steel compression platen at a constant rate of 0.1 mm/s until 0.6 to 1.2 N of force was exerted. Force measurements were taken 10 times per second using a 10-N load cell (Instron). Hook sizes were then measured under a microscope and correlated to the applied force.

### Tissue retention experiments

Fresh GI tissue was collected from euthanized swine and stored on ice. All ex vivo swine evaluations were conducted within 6 hours of euthanasia. Human specimens, which were shipped on ice in a buffer solution, were used for ex vivo experiments within 24 hours of the donor’s passing. Excess fat was trimmed from all tissue, and a 7 × 7 cm^2^ or larger portion of tissue was fixed in place by pushing pins into a piece of corkboard (McMaster Carr, Elmhurst, USA) firmly attached to a 6-mm-thick acrylic sheet (McMaster Carr, Elmhurst, USA). A hole with a diameter of 25 mm was previously cut through the center of the corkboard and the acrylic plate to allow the tissue to stretch vertically (fig. S4). A second piece of acrylic was added on top of the tissue to further fix the tissue in place when necessary.

To characterize the forces required to remove hooked needles from the tissue, these needles were fixed to the load cell of the Instron and lowered into a harvested piece of swine stomach or human stomach at a constant rate of 0.1 mm/s. Once they reached a predetermined displacement between 0.9 and 5 mm, they were backed out at a constant rate of 0.1 mm/s until the needles lost their hold on the tissue. During the hooking event, we measured the maximum distance the hooked needle pulled the tissue from its resting position as well as the force profile exerted on the needle. Once we determined the optimal displacement required to achieve the greatest hooking force, we tested needles with different hook lengths in the same manner.

To characterize the forces required to remove hooked needles from in vivo tissue and confirm the results from the ex vivo studies, hooked needles were fixed to a custom-made linear stage. We sedated female swine (65 to 85 kg) with intramuscular injection of Telazol (tiletamine/zolazepam) (5 mg/kg), xylazine (2 mg/kg), and atropine (0.05 mg/kg). Animals were intubated and maintained under anesthesia with isoflurane (1 to 3% in oxygen). A ventral midline laparotomy was performed to access the stomach. A 3- to 10-cm incision was made in the stomach wall to access the gastric mucosa. A portion of the gastric mucosa was stabilized, and needles were then lowered into a revealed portion of swine gastric tissue at a constant rate of 0.2 mm/s using a custom-made stepper motor attached to a force gauge. Once they reached a predetermined displacement, they were raised at a constant rate of 0.2 mm/s until the needles lost their hold on the tissue. Needle placements were random and spread out throughout each swine stomach. Force measurements were taken using a 10-N FG-3000 digital force gauge (Shimpo, Glendale Heights, USA) attached to the linear stage. Measurements were read using its accompanying software. During the hooking event, we measured the maximum distance the hooked needle pulled the tissue from its resting position as well as the force profile exerted on the needle.

The needles with hook lengths that generated the maximum hooking force were then implemented into the STIMS actuation system. We fixed a varying number of such needles to the device’s central spring, and we measured the retention forces created by these needle configurations after they were implanted into tissue. Once the device was assembled, the STIMS device containing one to three needles was placed on a freshly harvested piece of swine stomach tissue measuring 7 × 7 cm^2^ or larger. The needles were penetrated into the tissue, and the tissue was mounted onto a metal plate. This metal plate was fixed perpendicularly to the base of the Instron machine and positioned directly below a bar attached to a 500-N load cell (Instron) on the Instron. The bar was programmed to move at 0.1 mm/s. The bar pushed against the STIMS, and the force exerted was recorded until the STIMS was dislodged. This force and the gravitational force felt by the STIMS were summed to yield the retention force achieved by the inserted needles. In addition to measuring the retention force with varying numbers of needles, we also measured the retention force with needles placed in contact and up to 1.5 mm apart from each other. To ensure these distances were accurate, acrylic sheets were laser cut using a ULS VLS6.60 class 4 laser (Universal Laser Systems, Scottsdale, USA) with holes at predetermined gap lengths. The needles were inserted into these holes while they were fixed into the devices. STIMS devices with three needles placed 1 mm apart were then tested for retention in vivo and compared with ex vivo experiments. Devices were randomly placed on the exposed swine stomach tissue of a sedated swine, and the tissue was rotated 90°. The digital force gauge was then used to measure the retention force for the device. The batteries and microcontroller were not included in the devices used in this experiment because the force experienced by gravity for the batteries and microcontroller (~5 mN) was over one order of magnitude less than the retention force measured in the retention studies for the device with an array of three hooked needles. Gravitational forces were factored into the calculations after performing the experiments.

To characterize needle insertion forces and penetration events, we used the same setup as described above for the hooking force experiments in which we lowered needles at a constant velocity into a fixed piece of tissue. Penetration events were defined as inflection points in the force versus displacement characterization graph (fig. S8). Muscular tissue perforation was defined by visualization of the needle tip passing through the entire tissue thickness and only occurred after the last penetration event. To approximate the needle penetration depth after the initial penetration event, we incorporated tissue marking dye into the needles. We visualized that the dye passed completely through the mucosa after this penetration event by manually separating the outer muscular layer from the mucosa.

Orally delivered STIMS devices were confirmed by endoscopic visualization to attach and anchor to the stomach. Simulated ambulation confirmed that the devices remained attached to the stomach tissue even in the presence of motion. To determine the anchoring force of these devices, a laparotomy was performed after administration and actuation. A force gauge was then used to measure the exact force required to dislodge the devices.

### In vivo testing to verify electrical stimulation

To mimic the FDA-authorized Enterra gastric electrical stimulation system, a preprogrammed controller produced two consecutive pulses, with 70 ms in between, with a pulse length of 330 μs. The stimulation procedure was repeated every 5 s (*24*). Other devices with longer pulse lengths of 30 ms have also been reported for gastric stimulation in patients (*26*), and these stimulation times were also programmed into the microcontroller to demonstrate the versatility of the system. However, the STIMS studies characterizing the current and voltage passed through the tissue focused on the shorter pulse of 330-μs length.

We used a PIC10(L)F322 microcontroller (Microchip, Chandler, USA) in our STIMS device, which offered PWM to control both the pulses and the time between the sets of pulses. It was also selected for its small size. This allowed it to easily fit within an ingestible 000 capsule along with two Energizer 1.55-V silver-oxide coin batteries (Digikey, Thief River Falls, USA), the self-orienting system, and stranded core wires used to connect the electronics together (Digikey). We programmed this microcontroller in MPLAB X Integrated development Environment (Microchip Technology, Chandler, USA) and used a PICkit 3 In circuit Debugger (Microchip Technology) as the debugger and programmer. A circuit was created connecting the microcontroller to the batteries, the needles, and a TDS 2012c oscilloscope (Tektronix, Beaverton, USA) for measuring output current and voltage. The circuit was left open between the two needles to allow the circuit to be completed by the tissue. Devices were assembled up to 1 day before the in vivo experiments and maintained their ability to produce electrical pulses.

We performed in vivo experiments on swine stomach to evaluate the performance of the electronic setup in the complex and dynamic gastric environment. Detailed sedation and surgical procedure methods can be found in the “Tissue retention experiments” section. Once a working area of gastric tissue of at least 80 mm by 80 mm was revealed, the electric probes were positioned 4 mm apart and inserted into the tissue from the mucosa into the muscular layer in each of the following areas of the GI tract: stomach, small intestine, and colon. The circuit created is shown in Fig. 1. Needles were positioned 4 mm apart because this was the largest allowable distance possible between two needles based on the device design. Making the exit hole any larger to allow a further needle separation inhibited the ability of the device to self-orient.

We measured the voltage over time using the TDS 2012c oscilloscope. Voltage measurements were taken over the entire circuit (to get values for Fig. 4G), and they were also taken over a 10-ohm resistor connected in series with the tissue to quantify the current running through the system. The current passing through the circuit was calculated by dividing the measured voltage by the known resistance in the resistor. To eliminate noise, a total of 16 pulses were averaged in the calculation. The maximum charge density at the electrode-tissue interface was calculated as the total charge (*Q*) divided by the cross-sectional area of the lead in contact with the tissue (S) (Eq. 1) (*26*)

$$\frac{Q}{S}=\frac{I\times T_{\text{phase}}}{\pi \mathrm{LD}}=\frac{5\times 0.33}{\pi \times 1\times 0.235}=2.2\frac{\mu C}{{\text{cm}}^{2}}$$ (Equation 1)

In this equation, *I* is the pulse amplitude in mA, *T*_phase_ is the pulse width in ms, *L* is the length of the exposed lead, and *D* is the diameter of the lead.

Muscular stimulation was imaged using a Sonoscape S9 ultrasound imaging machine (Universal Imaging, Belford Hills, USA).

For orally administered STIMS devices, it was not possible to calculate the exact magnitude of the electrical pulse signals delivered to the local stomach tissue or capture acute muscle contractions because the oscilloscope and ultrasound imager could not access the stomach via the overtube. However, device attachment was confirmed via endoscopic visualization, and the electrical pulses passing through the tissue were measured using the conductive communication method described below.

### Conductive communication

Two STIMS probes were placed into a euthanized swine’s stomach muscle or gastric lumen, or placed outside of the body. These probes were connected to an Arduino Genuino Uno (Digikey) programmed to produce two consecutive 5-V pulses, with a pulse length of 330 μs. While an Arduino was used during these proof-of-concept experiments to facilitate testing and recording over a wide range of pulse values (Fig. 5), we also demonstrated that the same conductive communication protocol could be used to read out the electrical pulses from an orally administered STIMS system containing a PIC10(L)F322 microcontroller (fig. S9). When using the orally administered STIMS system in these experiments, two of the probes were connected to the microcontroller while the third probe was used solely to anchor the device. In this experiment, we alternated the pause time in-between pulses to ensure that the signal we recorded was not a physiological signal or a signal from the background. Multiple pause times were tested from 0.5 ms to 70 ms to 5 s, and multiple pulse lengths were tested with pulses ranging from 1 μs to 30 ms. We reported the data from the 330-μs pulse length, as this mimicked the pulse already produced for electrical stimulation. For the data in Fig. 5, we report the data from pulses delivered with alternating pauses of 0.5 and 1 ms. These pulses were closer together than the electrical stimulation procedure described for gastric pacemakers, and this allowed us to more easily capture multiple pulses on the oscilloscope. However, we confirmed that the PWM described for gastric pacing was also able to communicate stimulation events through a change in the voltage readout in the oscilloscope, as seen in fig. S9. Two 32-gauge uncoated needles were inserted 1 cm apart subcutaneously just below the swine’s abdomen. These needles were interconnected by a 10-ohm resistor for the data presented in Fig. 5, and the resistor was not present when collecting the data for fig. S9. The presence of the resistor allowed for current to be measured; however, removing the resistor accentuated the differentiation between the peak voltage measured by the oscilloscope during stimulation compared with baseline measurements without stimulation. The current passing through the body was measured by determining the voltage drop over this resistor. The voltage drop was measured using a Rigol DS1054 Oscilloscope (Rigol, Beaverton, USA). During each measurement, the probes in the stomach and in the subcutaneous space were moved to another spot in the stomach and subcutaneous space, respectively. The average current passed through the circuit branch during one pulse was then measured and plotted. The same procedure was repeated, except this time, the pulse was delivered to the subcutaneous space and the voltage drop was recorded in the stomach. Notably, the instrumentation used to conduct these studies was in parallel with the STIMS device’s circuit. The reason this is important is because the current that passes through the branch of the parallel circuit that goes to the oscilloscope is always less than or equal to the total current supplied by the STIMS power source, as some of the current passes through a different branch instead.

### Synthetic stomach assembly

A recirculating flow system was assembled using an inline circulation pump (McMaster) with a flow rate of 9 liter/min and Tygon tubing (McMaster-Carr) ranging from 13-mm diameter at the outlet up to 50 mm in diameter in the tissue chamber. The flow rate was adjusted to the preferred rate using a pressure regulating valve (McMaster). A piece of stomach tissue was fixed to the Tygon tubing using staples, and the STIMS devices were then inserted into the tissue at 50-mm intervals. Devices were placed in triangular patterns, which prevented disruptive eddies from affecting those downstream. The tissue tube was oriented so that the devices and tissue were situated perpendicular to the gravitational force. The circulation system was connected to a large, 75-liter water reservoir that filtered out any tissue particles that entered the flow. The pump was then connected to an Arduino Uno microcontroller, which was programmed to generate pulsatile flow in the system by turning the pump on and off every 15 s. The entire setup was run for a total of 7 days, and devices were checked daily to verify retention. The batteries and microcontroller were not included in the devices used in this experiment because the force experienced by gravity for the batteries and microcontroller (~5 mN) was over one order of magnitude less than the retention force measured in the retention studies for the device with an array of three hooked needles.

### Statistical analysis

No data were excluded from the analysis. Student’s *t* tests were performed using Prism Version 7.0 (GraphPad) or Microsoft Excel (Microsoft). A value of *P* < 0.05 was considered statistically significant. Figure captions describe the number of replicates used in each study. Figure captions define the center line and error bars present in the plots.

## Supplementary Material

aaz0127_Movie_S4.mp4

aaz0127_Movie_S3.mp4

aaz0127_SM.pdf

aaz0127_Movie_S2.avi

aaz0127_Movie_S1.mp4

## SUPPLEMENTARY MATERIALS

Supplementary material for this article is available at http://advances.sciencemag.org/cgi/content/full/6/35/eaaz0127/DC1

## Appendix

View/request a protocol for this paper from *Bio-protocol*.
