# Supplementary material for: Ingestible transiently anchoring electronics for microstimulation and conductive signaling
Source: Sci Adv. 2020 Aug 28;6(35):eaaz0127. doi: 10.1126/sciadv.aaz0127 (PMC7455191; doi:10.1126/sciadv.aaz0127)
Supplement: aaz0127_SM.pdf [file aaz0127_SM.pdf]

Science Advances
AAAS

advances.sciencemag.org/cgi/content/full/6/35/eaaz0127/DC1

Supplementary Materials for

Ingestible transiently anchoring electronics for microstimulation and conductive signaling

Alex Abramson, David Dellal, Yong Lin Kong, Jianlin Zhou, Yuan Gao, Joy Collins, Siddartha Tamang, Jacob Wainer, Rebecca McManus, Alison Hayward, Morten Revsgaard Frederiksen, Jorrit J. Water, Brian Jensen, Niclas Roxhed, Robert Langer*, Giovanni Traverso*

*Corresponding author. Email: rlanger@mit.edu (R.L.); cgt20@mit.edu (G.T.)

Published 28 August 2020, *Sci. Adv.* **6**, eaaz0127 (2020)
DOI: 10.1126/sciadv.aaz0127

The PDF file includes:

Figs. S1 to S9
Table S1

Other Supplementary Material for this manuscript includes the following:

(available at advances.sciencemag.org/cgi/content/full/6/35/eaaz0127/DC1)

Movies S1 to S4

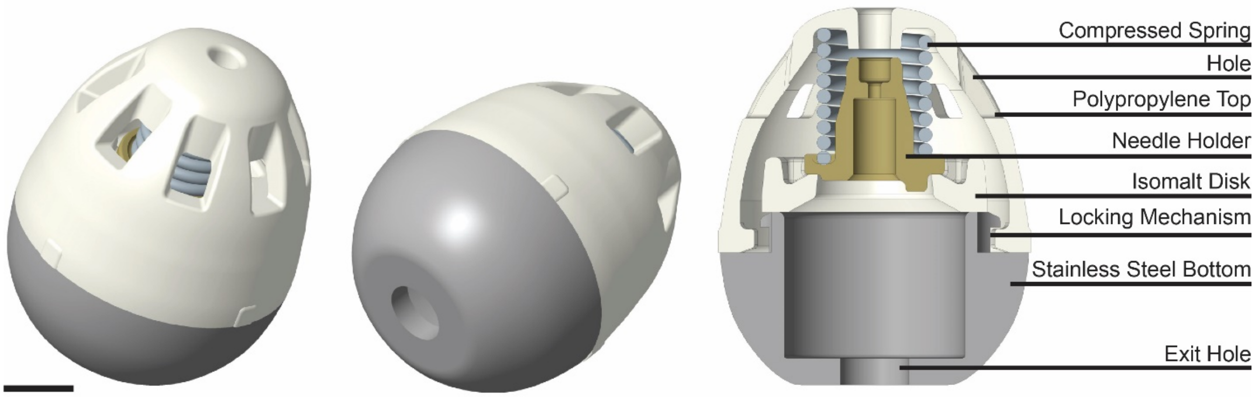

Supplementary Fig. S1: STIMS device design. The top portion of the device, fabricated from plastic, possessed holes which allowed fluid to enter and dissolve the actuating isomalt disk (seen in Supplementary Fig. S2). The small size of the holes prevented rapid fluid flow through the device and slowed the dissolution of the isomalt disk. By changing the thickness of the isomalt disk and device hole size, we were able to define the actuation time of the system. Once the isomalt disk dissolved, the compressed spring expanded and accelerated the needle holder. The needle holder pushed the needles (not shown) out of the exit hole. The stainless steel bottom shifted the center of mass towards the preferred orientation, which allowed the device to self-orient. The rotational locking mechanism kept the bottom and top pieces together even after the spring actuated. After actuation, the spring was stopped by the stainless steel bottom and did not exit the device. By increasing the radius of the open hole in the stainless steel bottom, we were able to increase the needle displacement distance. (Scale bar = 2 mm).

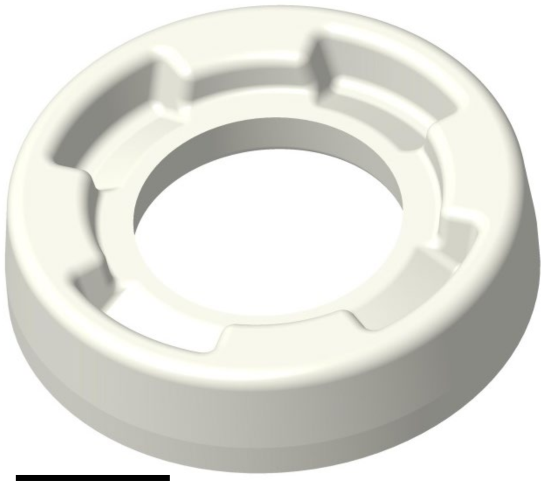

Supplementary Fig. S2: Isomalt disk design. The isomalt disk was used to hold and actuate the compressed spring. The spring was held in compression until it was released after the isomalt dissolved. Holes in the top of the device allowed fluids to enter and dissolve the isomalt disk at a controlled rate. The depressions in the disk ensured that the disk broke in specific sections. The areas with depressions dissolved more quickly than other areas which created preferentially weak spots in the disk. By breaking in specific spots, we ensured that the isomalt disk did not block the path of the spring after actuation. Instead, it broke into a few small pieces which were pushed aside by the expanding spring. The isomalt disk broke and actuated the device approximately 5-15 minutes after the device was placed in warm water or in the digestive tract of a sedated swine, but this actuation time can be tuned based off of the thickness and shape of the disk as well as the size of the holes in the device top. (Scale bar = 2 mm).

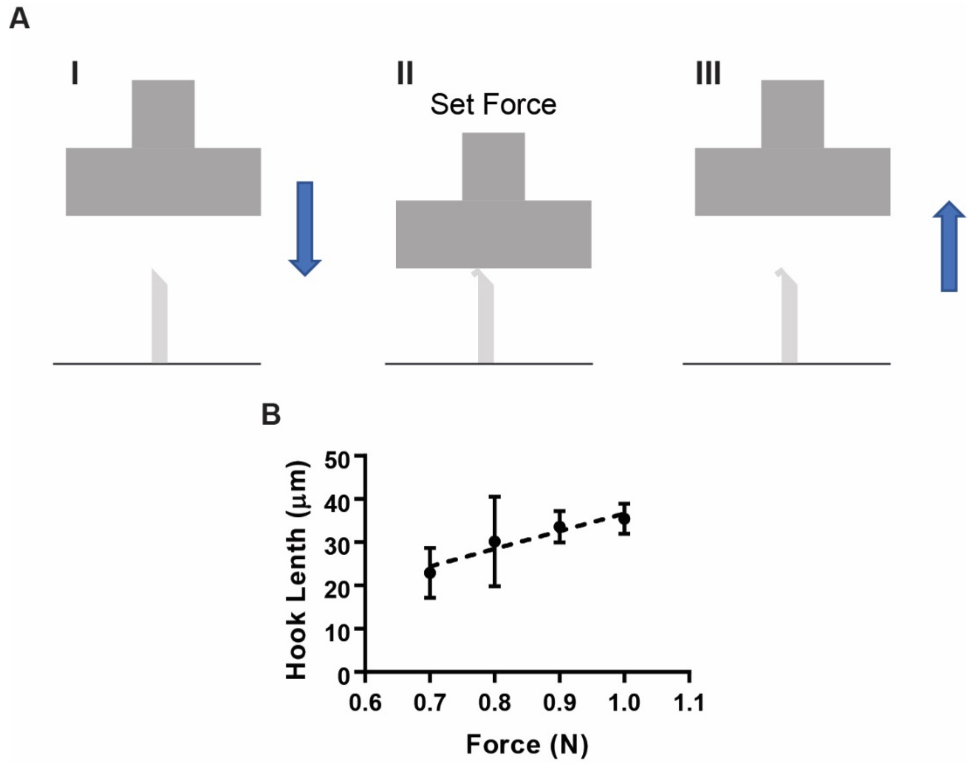

Supplementary Fig. S3: Hooked needle fabrication. (a) Using a flat stainless steel compression platen attached to an Instron machine, we apply a set force to the tip of the needle to create a hook. (b) The hook length is defined by the force applied to the needle tip. (n=3). Error bars = SD

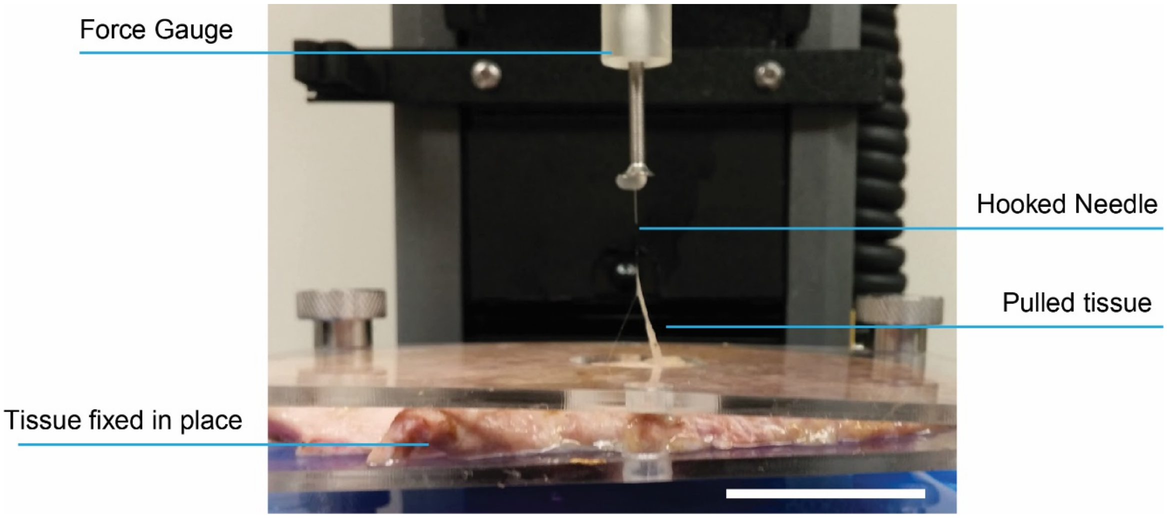

Supplementary Fig. S4: Hooked needle testing. We utilized an Instron machine to test the penetration forces and the hooking forces of the needles. The tissue was fixed in place and pinned down at its corners without adding tension. We cut a hole in the middle of the acrylic and corkboard sheets in order to allow the tissue to stretch freely in the vertical direction. The tissue pictured here is swine tissue. (Scale Bar = 5 cm). Photo Credit: Alex Abramson, MIT.

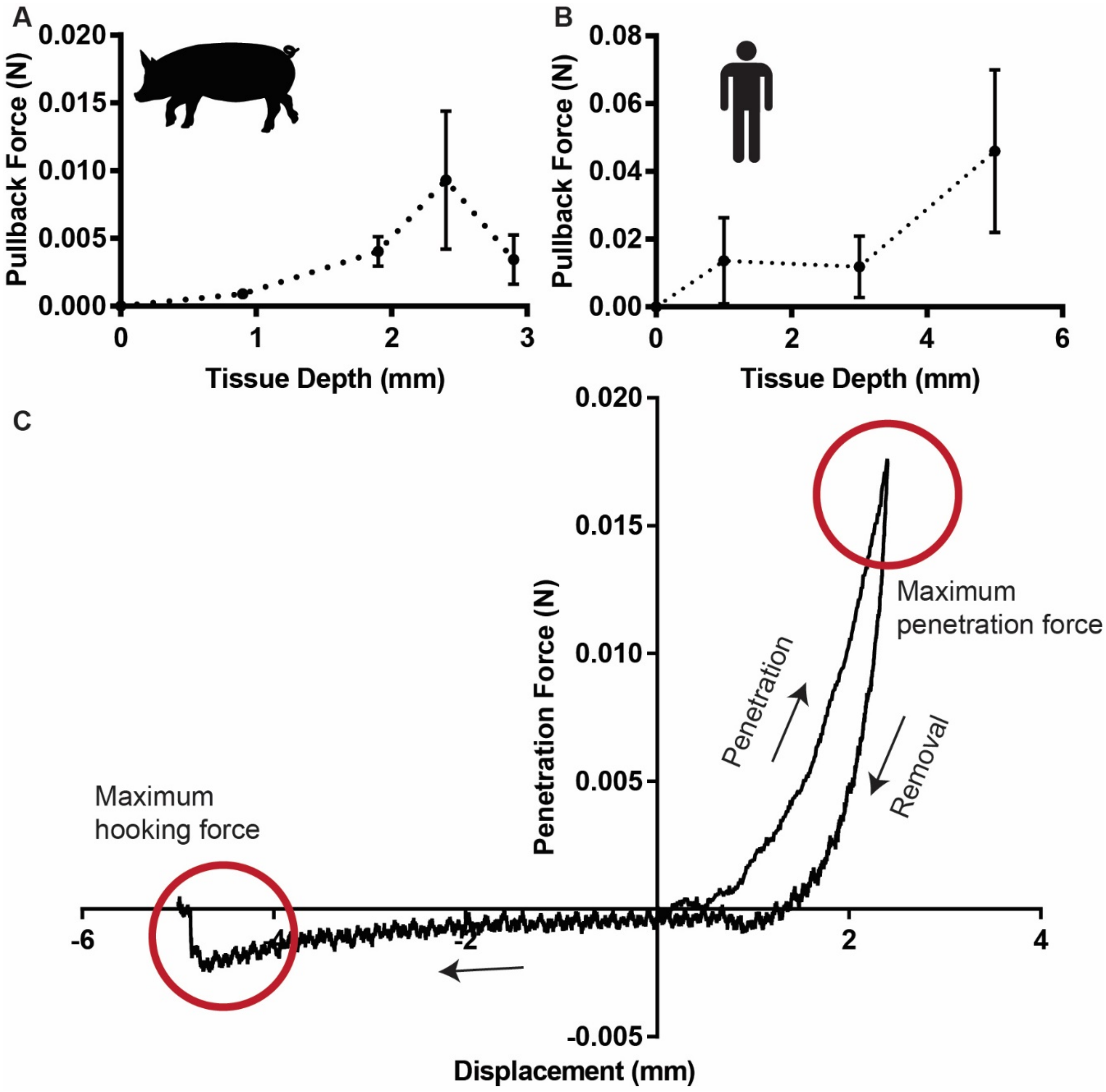

Supplementary Fig. S5: Hooking forces in swine and human by depth. Hooked needles in (a) swine and (b) human stomach tissue only begin to attach to the tissue after a defined insertion depth. (n=3). (c) A typical force graph of a hooked needle inserted and removed from swine stomach tissue. Error bars = SD.

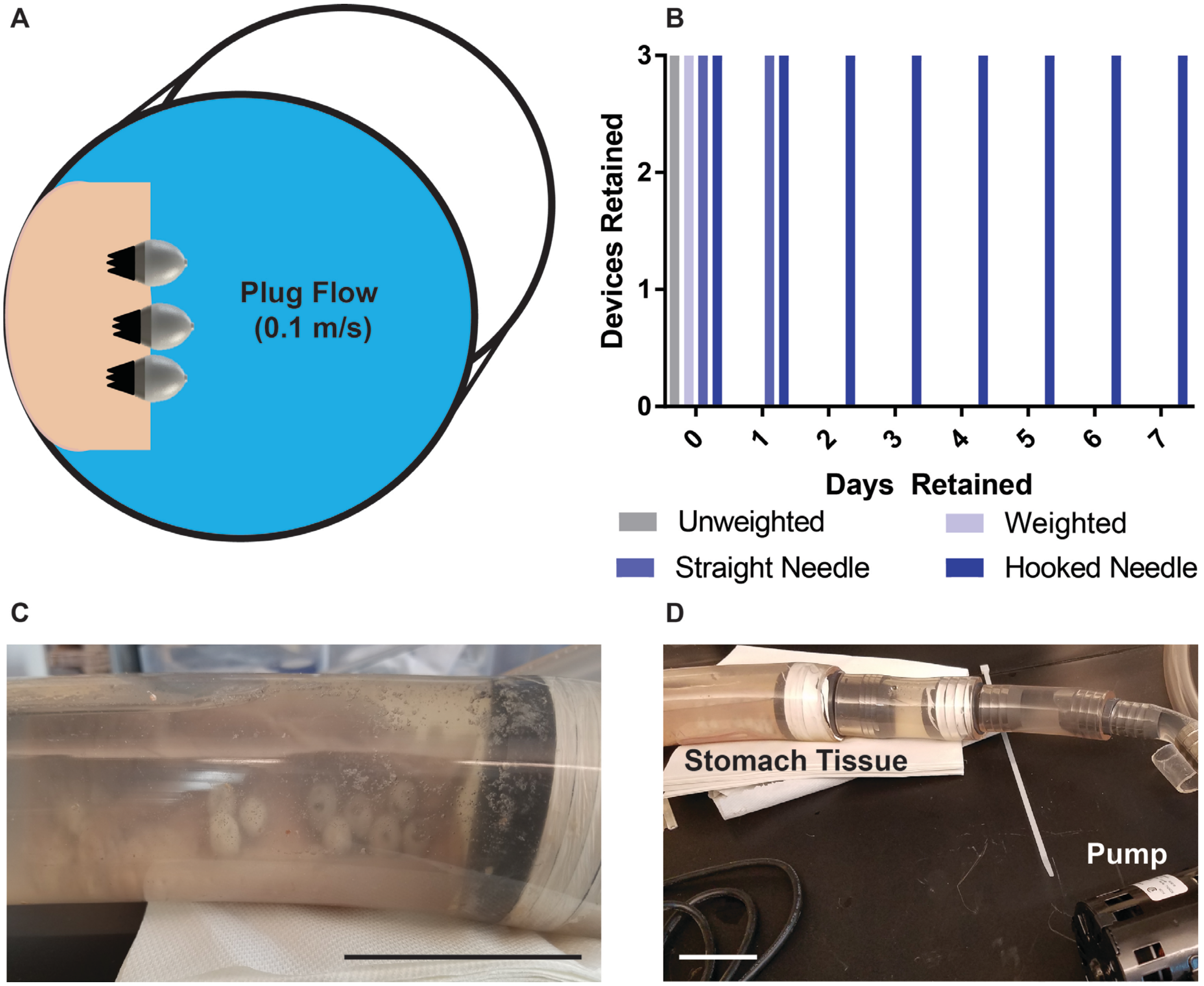

Supplementary Fig. S6: Horizontal *ex vivo* retention. (A) We placed STIMS devices on stomach tissue suspended in a pipe perpendicular to the gravitational force. An animation of the synthetic stomach is provided. (B) Unweighted and weighted STIMS devices without needles fell off the tissue immediately within seconds to minutes. Unweighted devices were also washed away from their initial position in the system. STIMS devices with needles inserted in the tissue remained attached to the tissue. To simulate gastric flow, we pumped a turbulent flow of water at 0.1 m/s though the pipe over the course of 7 days and monitored retention on the tissue. The STIMS devices with unmodified needles fell off within 1 day while the STIMS devices with hooked needles remained attached for 7 days and showed no sign of imminent detachment (n=3). (C,D) Images of the synthetic stomach system using *ex vivo* swine tissue and a pump to mimic fluid flow in the stomach. Batteries and microcontrollers were not attached to the devices during this test, because the gravitational and drag forces felt by the electronics were orders of magnitude less than the retention force generated through the needles. (Scale Bars = 5 cm). Photo Credit: David Dellal, MIT.

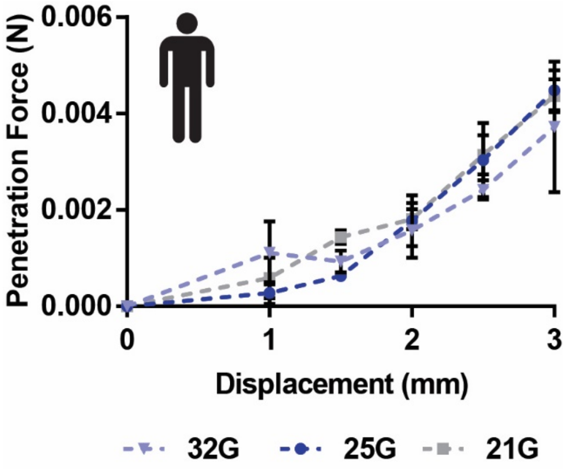

Supplementary Fig. S7: Penetration forces in a human stomach using various needle gauges. Using various needle gauges does not change the penetration forces required to displace the needle into the tissue up to 3 mm (n=3). Error bars = SD.

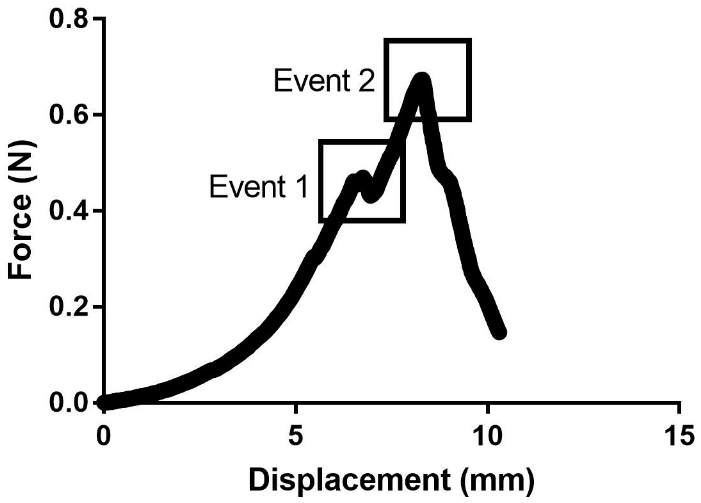

Supplementary Fig. S8: Force versus needle displacement curve of a 21G hypodermic needle penetrating through swine stomach tissue *ex vivo*. There are two distinct penetration events, defined as inflection points on the graph. Event 2 represents perforation of the muscular tissue, defined by the visualization of the needle tip passing completely through the tissue. After event 1, there was no visualization of the needle tip on the other side of the tissue, and therefore perforation of the muscular tissue did not occur. To approximate the depth of needle penetration at event 1, we coated the needle in tissue marking dye and manually separated the mucosa from the outer muscular layer of the stomach after the first event but before the second event. After separating the tissue, we visualized dye which passed completely through the mucosa of the tissue.

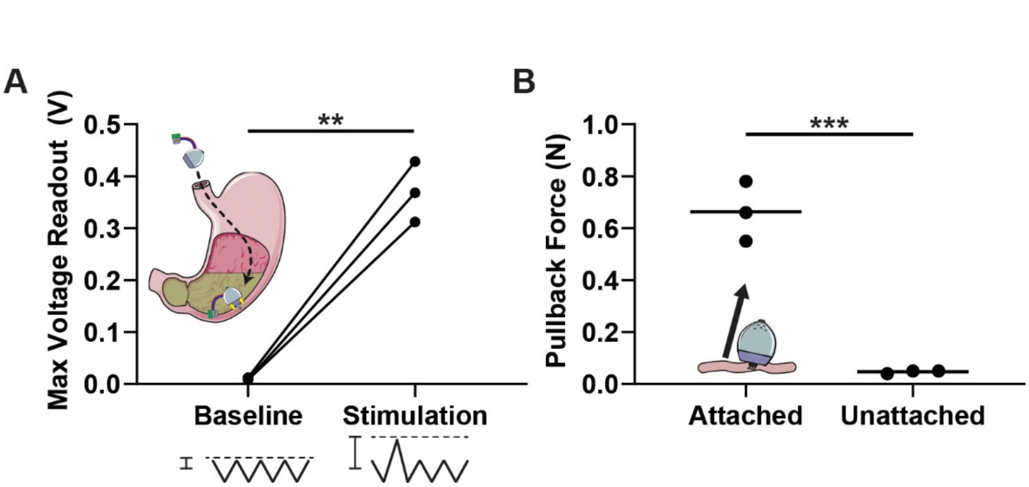

Supplementary Fig. S9: STIMS administration via an overtube to in vivo swine models. (A) Conductive communication confirmed devices provided electrical stimulation after device administration via an overtube, self-orientation and actuation in the stomach. The peak voltage measured by the oscilloscope during stimulation was compared to baseline measurements without stimulation. (n=3 devices). (B) Device retention was confirmed by comparing the maximum force required to remove attached (hooks + gravity) devices from the stomach tissue to the maximum force required to lift unattached (gravity alone) devices. (n=3 devices). (Line = Mean. **P<0.01; ***P<0.001).

	Age	Sex	Race	Height (m)	Weight (kg)	Mode of Death
Patient 1	66	F	C	1.68	81.2	Cerebrovascular/stroke
Patient 2	55	M	C	1.85	86.2	Cardiac/respiratory
Patient 3	63	F	C	1.63	58.1	Cardiopulmonary Arrest

Supplementary Table S1: Human patient data from stomach penetration study. Characteristics of the patients from which the stomachs were acquired.
